# Valorization of Aquatic Weed and Agricultural Residues for Innovative Biopolymer Production and Their Biodegradation

**DOI:** 10.3390/polym13172838

**Published:** 2021-08-24

**Authors:** Prapaipan Ungprasoot, Papasanee Muanruksa, Varavut Tanamool, James Winterburn, Pakawadee Kaewkannetra

**Affiliations:** 1 Graduate School, Khon Kaen University, Khon Kaen 40002, Thailand; un_prapaipan@kkumail.com; 2 Research Center for Environmental and Hazardous Substance Management (EHSM), Faculty of Engineering, Khon Kaen University, Khon Kaen 40002, Thailand; m.papasanee@kkumail.com; 3 Chemistry Program, Faculty of Science and Technology, Nakhon Ratchasima Rajabhat University, Nakhon Ratchasima 30000, Thailand; varavut.t@nrru.ac.th; 4 Department of Chemical Engineering and Analytical Science (CEAS), The University of Manchester, Manchester M13 9PL, UK; James.Winterburn@Machester.ac.uk; 5 Department of Biotechnology, Faculty of Technology, Khon Kaen University, Khon Kaen 40002, Thailand

**Keywords:** valorization, water hyacinths, sugarcane bagasse, rice straw, biodegradation

## Abstract

In this work, water hyacinths, bagasse and rice straw were valorized to produce an innovative biopolymer. Serial steps of extraction, bleaching and conversion of cellulose to be carboxymethylcellulose (CMC) as well as the last steps of blending and molding were performed. The CMC was mixed with tapioca starch solution by a ratio of 9:18, and a plastic sizer of glycerol was varied at 2%, 4% and 6% by volume. In addition, bioplastic sheets were further determined in their properties and biodegradation. The results revealed that bioplastics with 6% glycerol showed a high moisture content of 23% and water solubility was increased by about 47.94% over 24 h. The effect of temperature on bioplastic stability was found in the ranges of 146.28–169.25 °C. Furthermore, bioplastic sheets with 2% glycerol could maintain their shape. Moreover, for texture analysis, the highest elastic texture in the range of 33.74–38.68% with 6% glycerol was used. Moreover, bioplastics were then tested for their biodegradation by landfill method. Under natural conditions, they degraded at about 10.75% by weight over 24 h after burying in 10 cm soil depth. After 144 h, bioplastics were completely decomposed. Successfully, the application of water, weed and agricultural wastes as raw materials to produce innovative bioplastic showed maximum benefits for an environmentally friendly product, which could also be a guideline for an alternative to replace synthetic plastics derived from petroleum.

## 1. Introduction

Currently, the world is facing various environmental pollution problems. According to economic expansion, synthetic plastic derived from petroleum has increased because of its low cost of production and durability. However, it is disposed of in large quantities in the environment. Over the last 10 years in Thailand, the amount of synthetic plastic waste has increased by 2 million tons per year, and this waste has had a huge impact on the environment, is difficult to remove and cannot be decomposed naturally [1]. It could take up to 450 years to decay. Therefore, bioplastics can be a solution to these environmental problems. The raw materials used in production can be obtained from nature and have natural decomposition properties. Currently, bioplastics, made from a polymer of natural acids, are produced by microorganisms through the fermentation process. For instance, polyhydroxyalkanoates (PHAs), polyhydroxybutyrate (PHB), Polylactic acid (PLA), etc. Bioplastics can also be produced from aquatic weeds and agricultural wastes that can be obtained from agricultural residues, the wood industry and livestock waste. Most of them are crops that can be rotated, planted and replaced in a short period of time, such as corncobs, sugarcane bagasse, cassava, rice straw, water hyacinth, palm bunch, etc. [2,3,4]. These waste materials are disposed of to decompose by influences from physical, chemical and biological factors. There are natural mechanisms for enzymatic or bacterial biodegradation under proper temperature and humidity. These natural decomposition products include water, biomass, methane and carbon dioxide, which are important for the growth and sustenance of plants.

Biomass waste is an environmentally friendly renewable resource. It is classified as a natural carbon source, which mainly contains cellulose, hemicellulose, lignin, chitin, ash and proteins. Thus, it is particularly suitable to be used as a precursor to prepare innovative carbon-based materials [5]. Recently, biocomposite plastic was developed by blending bioplastic with biomass. Previously, Rodion Kopitzky [6] studied cost effective bio-based plastic production by blending polylactic acid-polybutyrene with succinate-sugar beet pulp particles. In addition, cellulose-based composite material production was also reported by Sun et al. [7]. They used cellulose powder, which was obtained from filter paper pulp, as raw material for cellulose hydrogel production.

Thermoplastic starch (TPS) is an environmentally friendly plastic that can be produced from various natural resources. It provides an advantage in terms of cleaner production, because its production process does not depend on petroleum oil [8,9,10]. Typically, cassava starch is widely used as renewable material for starch-based plastic production at the industrial scale [11]. However, there are some limitations of TPS due to its poor mechanical properties, high moisture adsorption and unstable structure that is caused by relative humidity [12,13]. Accordingly, one popular strategy to increase the stability of TPS is blending starch with plasticizer. Glycerol is a plasticizer that has high ability to improve the mechanical properties of starch-based plastic [14,15]. Previously, Chen et al. [14] investigated the effect of glycerol on corn starch morphologies and the gelatinization process under warm temperature (65 °C). The results showed that the addition of glycerol to corn starch can enhance the strength of plastic, and starch gelatinization was postponed to higher temperatures. Another strategy that has been used is blending starch with fibers, nanofibers and synthetic polymers to overcome the problems of retrogradation and the poor mechanical properties of starch-based plastic [16,17,18,19].

Therefore, water weeds and agricultural waste including water hyacinth, sugarcane bagasse and rice straw were used as natural fiber resources to produce bioplastics that maximize the potential benefits. The carboxymethylcellulose, which was obtained from waste biomass via extraction techniques, acted as innovative material for bio-based plastic production. It was further blended with two bioplasticisers (starch and glycerol) in order to form biocomposite plastic. In addition, its biodegradable characteristic was also investigated by soil burial test. The target of this study was to generate bioplastic which can be naturally decomposed, leading to reduction of pollution from petroleum-based plastics. Finally, applications of biocomposite plastics were also evaluated from this characteristic.

## 2. Materials and Methods

### 2.1. Preparation of Raw Materials

Hyacinth, an aquatic weed, was collected from natural canals and water basins. It was washed with clean water to remove soil attached at its root. Then, it was cut to a size of about 1 × 1 cm and then dried by sunlight for 3 days. Meanwhile, agricultural wastes of sugarcane bagasse and rice straw were also collected from onsite at sugarcane farms and rice fields in Khon Kaen province and nearby provinces. The wastes were then sliced, cut and ground to a small size. Other chemicals used were of analytical grade and purchased from Merk Chemical Co., Ltd., Bangkok, Thailand.

### 2.2. Cellulose Extraction and Bleaching

All prepared raw materials, water hyacinth, sugarcane residue and rice straw, were extracted to obtain cellulose by adding the sample into 1 M sodium hydroxide (NaOH) solution (15 times by weight of sample). Then, the pulp sample was heated on an electromagnetic stirrer at 90 °C for 30 min, and then it was filtered by thin white cotton cloth. The extracted cellulose was obtained. However, the cellulose sample was rinsed with clean water to remove sodium hydroxide solution until the pH of the sample reached 7. Finally, cellulose pulp obtained from water hyacinth, bagasse and rice straw was bleached with a mixed solution of 35% hydrogen peroxide, 2% sodium silicate and 0.05% magnesium sulphate by sample weight. The pulp pH was adjusted again to be pH 7.0 with one molar of NaOH and then heated at 90 °C for 20 min. The pulp cellulose sample was washed in triplication with clean water, before being dried in a hot air oven at 80 °C for 24 h.

### 2.3. Carboxymethylcellulose Synthesis

The bleached cellulose was converted to carboxymethylcellulose (CMC) by using a mixture solution of 18 g chloroacetic acid (C2H3ClO2) and 350 mL isopropyl alcohol (C3H8O) and heated by an electromagnetic stirrer at 40 °C for 30 min. Then another 50 mL of NaOH with 40% by volume was also added. The pulp solution was continuously stirred at 40 °C for 60 min. The beaker was sealed with aluminum foil and left in the hot air oven at 55 °C for 2 h 30 min until stratification occurred between the liquid and the cellulose sediment. The translucent part was poured and washed off the cellulose sediment with 70% methanol solution for 70 mL. The mixture was left for 10 min. To wash off the isopropyl alcohol solution, the supernatant was rinsed. Then, 70% ethyl alcohol solution at about 100 mL was added into the sediment. This step was repeated 5 times, pure ethyl alcohol was also added at about 200 mL, and the clear portion was discarded. The carboxymethylcellulose (CMC) precipitate was cured in a hot air incubator at 55 °C for 24 h.

### 2.4. Bioplastic Forming

The procedure method was modified from a previous study [20]. A synthetic CMC powder (9 g) was dissolved in 200 mL of distilled water and heated to 90 °C by an electromagnetic stirrer for 30 min. Subsequently, tapioca starch solution (18% *w*/*v*) was added and continuously heated for 20 min. Glycerol was added with varying concentrations of 2%, 4% and 6% by volume when all mixtures were of homogeneous viscous consistency. They were then poured into a mold of 90 × 15 mm^2^ and dried with a hot air oven at 55 °C for 24 h.

### 2.5. Moisture Analysis

A bioplastic sample weighed approximately 100 g and was placed on an aluminum foil tray. The total weight was recorded (M0) and was then baked at 80 °C for 24 h. It was left to cool down in a desiccant chamber. The sample was weighed and the results recorded (M1), following the ASTM standard E104-02 [21]. The results of the moisture content analysis can be calculated from Equation (1) as follows:

(1) $$\%\mathrm{Moisture}=\frac{M_{0}-M_{1}}{M_{0}}\times 100$$

where: *M*_0_ = the total sample weight before drying, and *M*_1_ = the total sample weight after drying.

### 2.6. Water Solubility

A filter paper (Whatman No. 1) was prepared by drying in a hot air oven at 105 °C for 24 h. Then, it was cooled down in a desiccant chamber and weighed. Prior to use for filtration, the paper was soaked in distilled water. Meanwhile, a prepared bioplastic sample sheet (50 × 50 mm) was dried in a hot air oven at 65 °C for 24 h. Then, it was cooled down to obtain stable weight in a desiccant cabinet and weighed, and the results were recorded. The bioplastic sample sheets were immersed in 50 mL of distilled water, left at room temperature for 24 h while filtered with prepared paper, dried at 80 °C for 24 h and kept to cool down (room temperature) in a desiccator. Both of them were weighed and recorded as *W*_0_ and *W*_1_. The solubility analysis results from Equation (2) are shown as follows:

(2) $$\mathrm{Water}\;\mathrm{Solubility}\;\left(\%\right)=\frac{W_{0}-W_{1}}{W_{0}}\times 100$$

where: *W*_0_ = total weight before testing, and *W*_1_ = total weight after testing.

### 2.7. Thermal Property

For thermal property, bioplastics were tested in their stability by using Differential Scanning Calorimeters (Shimadzu DSC-60A, Kyoto, Japan) [22]. In brief, 10 mg bioplastic sample was prepared and placed in an aluminum dish and packed into the designated position inside the Differential Scanning Calorimeters device (DSC). The initial temperature was set at 10 °C to the final temperature of 200 °C. The temperature increase rate was 5 °C/min using nitrogen gas flow rate of 20 mL/min. All thermal measurements were performed on single sample and used for comparative purposes against the control sample.

### 2.8. Texture Analysis

For texture analysis by texture analyzer (CT3 Texture Analyzer, AMETEK, Berwyn, PA, USA), a bioplastic sample (3 cm × 5 cm) was cut, and then it was attached to the head and tail clamping arms of the machine. The velocity of the stretch probe was set at about 1.00 mm/s. The velocity of probe returning was set at 10.00 mm/s, while distance of the probe was touched at the point of impact (20.00 mm/s) milli. Then, stretch value of the texture and strength of the object were measured and recorded.

### 2.9. Biodegradation of Bioplastics

For biodegradation property test, 5 × 5 cm^2^ bioplastic samples were dried at 55 °C in hot air oven for 24 h and then were kept in desiccant chamber until achieving stable weight. The biodegradation test was started by burying the sample in 10 cm deep soil. The samples were weighed and recorded as sample coded as $D_{0}$. The sample, hereafter named as $D_{1}$, was withdrawn from the soil and cleaned once a day. The steps were repeated the same as the $D_{0}$ sample explained above. The sample’s weight loss over time was utilized to determine the biodegradation test in soil. The degradation rate in terms of percentage was calculated using Equation (3):

(3) $$\mathrm{Degradation}\;\mathrm{Rate}\;\left(\%\right)=\frac{D_{0}-D_{1}}{D_{0}}\times 100$$

where: $D_{0}$ = total weight before testing, and $D_{1}$ = total weight after testing.

## 3. Results and Discussion

### 3.1. Chemical Composition of Bioplastics

**Figure 1:**
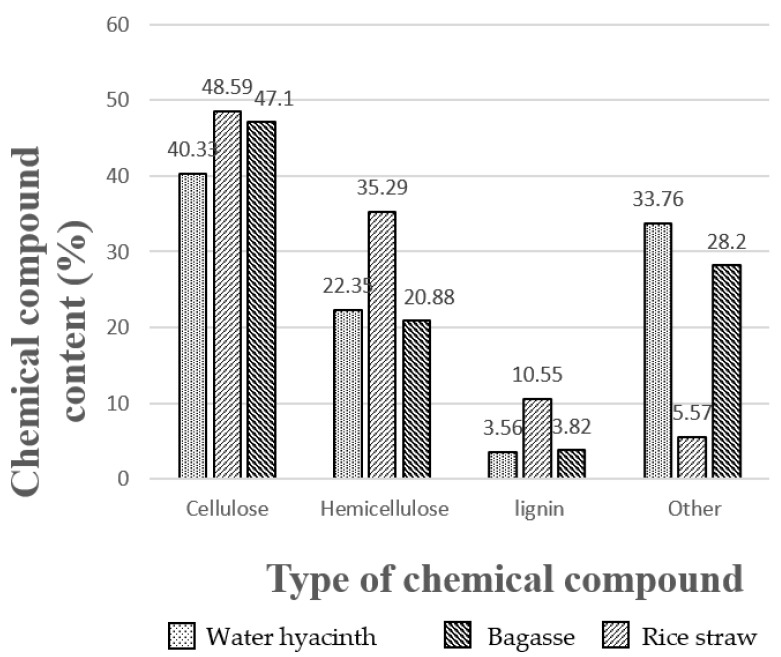
Chemical compositions of three different raw materials.

Chemical composition analysis of raw materials was measured by the fiber analysis technique (Detergent analysis) using a spectrophotometer. The quantitative analysis of cellulose, hemicellulose and lignin was detected. It was found that water hyacinth, sugarcane residue and rice straw at 100 g contained the quantitative cellulose of 40.33%, 48.59% and 47.10%, respectively (Figure 1). Cellulose pulp was extracted with NaOH solution and was bleached with a solution of hydrogen peroxide. Synthesis of carboxymethylcellulose was done with chloroacetic acid. For bioplastic forming, tapioca starch solution and glycerol were used as a binder at concentrations of 2%, 4% and 6% by volume as additive substances.

### 3.2. Texture Analysis

Texture properties of bioplastics with variable glycerol concentration show that when bioplastics had a high glycerol concentration of 6% by volume, it was found that at an average tensile strength in the range of 8–50 newtons, bioplastics from water hyacinth, sugarcane bagasse and rice straw showed a higher resilience rate of glycerol. The highest objects were 38.68%, 35.37% and 33.74%, respectively. Unlike bioplastics with low glycerol concentration at 2% by volume, it was found that bioplastics had low material resilience characteristics, and tearing easily accounted for 25.17%, 27.97% and 21.60%, respectively, from the texture analysis of all three types of bioplastics. It was shown that glycerol added to bioplastic samples showed additive properties and increased flexibility in bioplastic samples.

### 3.3. Thermal Property

**Figure 2:**
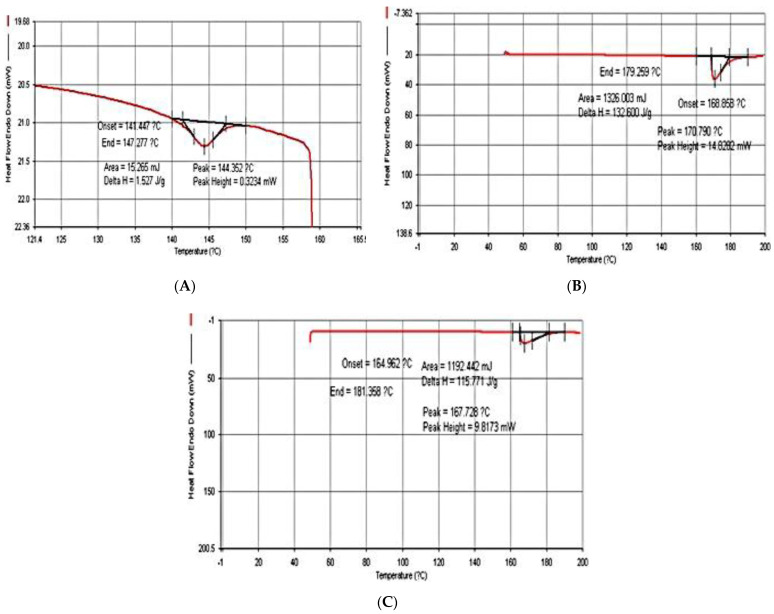
Bioplastic blended with 2% glycerol: (**A**) water hyacinth; (**B**) sugarcane bagasse; (**C**) rice straw.

**Figure 3:**
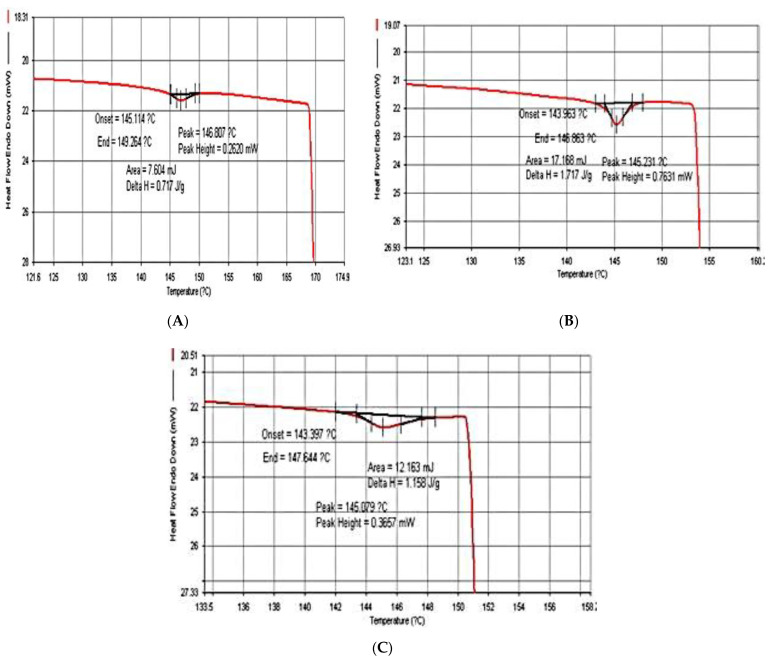
Bioplastic blended with 6% glycerol: (**A**) water hyacinth; (**B**) sugarcane bagasse; (**C**) rice straw.

The thermal characterization was completed by DSC experiments, the results of which are depicted in Figure 2 and Figure 3. It should be noted that the DSC results were shown only for 2 and 6% glycerol concentrations. For temperature analysis affecting the stability of bioplastics obtained from water hyacinth, sugarcane bagasse and rice straw, it was determined in terms of the melting point (*T*_m_) of the samples. It was found that when glycerol concentrations by volume were varied at 2, 4 and 6%, the maximal melting point in each sample was reached at 146.28 °C, 169.25 °C and 159.78 °C, respectively. Unlike bioplastics that were blended with 6% glycerol, the lowest melting points were 141.81, 145.74 and 146.08 °C, respectively, due to an increase in the moisture content of the samples caused by the insertion of the glycerol molecule in the bioplastic structure. Previously, the literature [5,6] reported that cellulosic bioplastic can be degraded at high temperatures (190–275 °C). According to results obtained in this work, the samples might easily lose their shapes at low temperatures and provide an environmentally friendly characteristic.

### 3.4. Water Solubility

Water solubility was determined to evaluate the effect on the decomposition of bioplastics derived from water hyacinth, bagasse and rice straw. It was found that water could absorb into all kinds of samples when 2%, 4% and 6% glycerol concentrations were used as shown in Table 1. Some physical changes in the samples were clearly observed such as swelling, deterioration and loss of morphological stability within 24 h, which was caused by the mixture of carboxymethylcellulose powder and tapioca starch. The starch powder, one of components in the bioplastic sample, showed good water solubility as glycerol concentrations increased up to 6%. Water solubility of 47.94% was found in accordance with the absorption properties of glycerol, though the samples could have absorbed moisture from the atmosphere. In addition, the sample sheet became soft and easy to decompose in a short time.

### 3.5. Moisture Analysis

**Figure 4:**
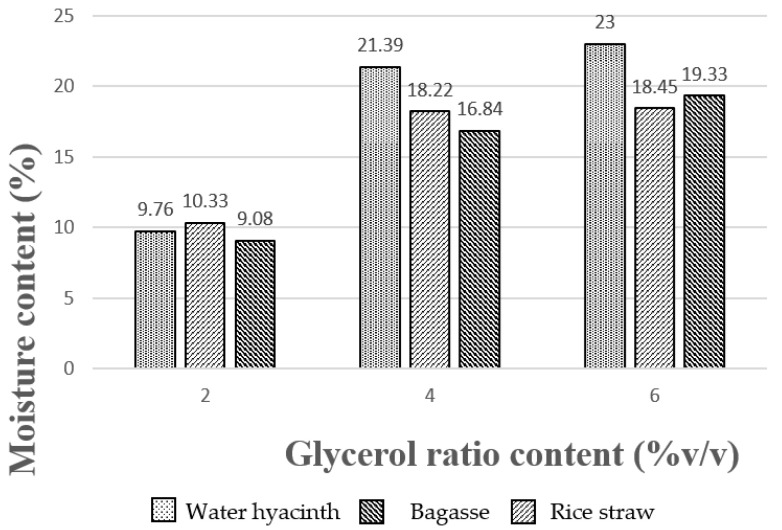
Moisture content of bioplastics obtained from three different raw materials.

The disadvantages of using glycerol as an additive in bioplastic samples at different concentrations resulted in high moisture content in all three bioplastic samples because glycerol is a hydrophobic compound that also has good absorbent properties in the atmosphere. Bioplastic samples from water hyacinth, sugarcane bagasse and rice straw with variable glycerol concentration at 2% by volume showed the lowest moisture content of 9.76%, 10.33% and 9.08%, respectively (see Figure 4). When the glycerol concentration used as an additive was increased to 6% by volume, all of the samples showed an increase in moisture content at 23%, 18.45% and 19.33%, respectively. Subsequently, it also caused more moisture absorption in the atmosphere.

### 3.6. Soil Burial Test

**Figure 5:**
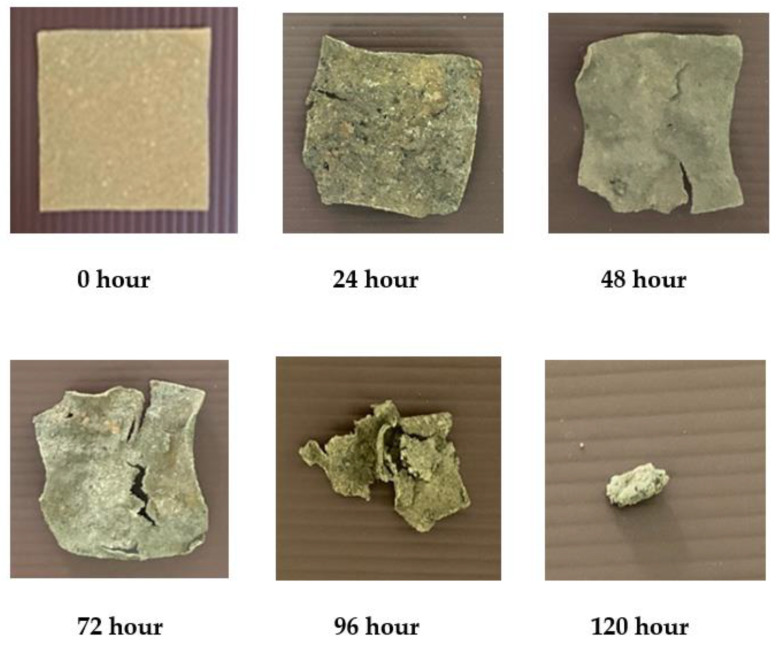
Burial test of bioplastic derived from water hyacinth at 6% glycerol.

**Figure 6:**
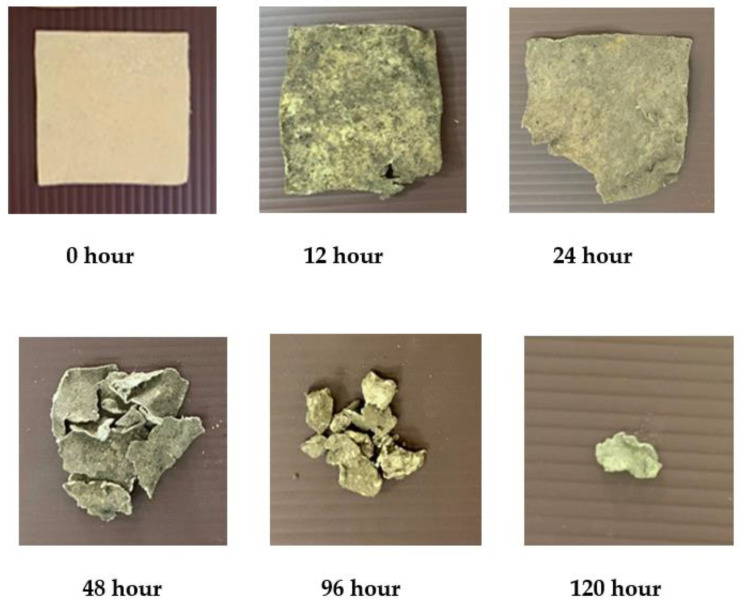
Burial test of bioplastic derived from sugarcane bagasse at 6% glycerol.

**Figure 7:**
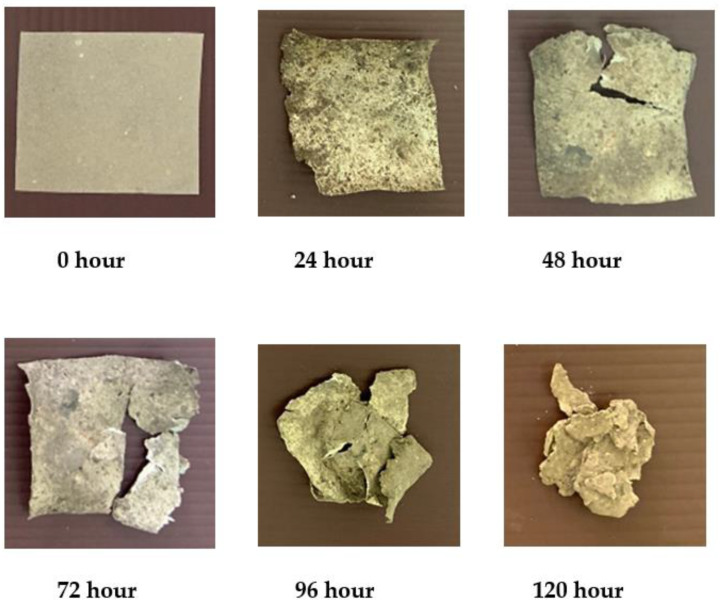
Burial test of bioplastic derived from rice straw at 6% glycerol.

Biodegradation of bioplastic samples from water hyacinth, sugarcane residue and rice straw by the landfill method was investigated under a natural environment. However, the burial test results were shown only for 6% glycerol concentration (see Figure 5, Figure 6 and Figure 7). The results showed that the physical appearance of the samples started biodegrading after 24 h. At 2% glycerol concentration, the degradation rate of bioplastic derived from water hyacinth, sugarcane residue and rice straw was 10.29%, 22.38% and 54.99%, respectively. They were continuously buried up to 120 h. Surprisingly, all of them were found to decompose completely, and no parts were present. This was perhaps caused by various microorganisms, such as soil fungi, which are starch-digesters, turning it into sugar for their growth. On the other hand, bioplastics blended with high glycerol concentration (4–6% glycerol concentrations) required 144 h to be completely degraded in the soil. These results agreed with a previous study [20], which investigated the biodegradation of biocomposite plastic via the soil burial method. The bioplastic sample was made from water hyacinth, starch and glycerol. It was found that 5% glycerol-added biocomposite samples showed a significant weight loss after 15 days, while 20% glycerol-added biocomposite samples presented lower weight loss and took more time for decomposition. This was due to starch and glycerol preventing the decomposition process catalyzed by microorganisms in the soil.

## 4. Conclusions

Successfully, water hyacinth, sugarcane residue and rice straw are capable as raw materials to convert into carboxymethylcellulose (CMC) and then use for biopolymer production, using tapioca starch solution and glycerol as a binder and an additive. The samples of biopolymer produced were characterized and showed the qualities of a synthetic plastic: an average texture tensile strength of 8 to 50 newtons, the highest flexibility at 38.68% and a melting point of 169.25 °C. In addition, for the decomposition test by water solubility, biopolymers showed an ability to decompose within 24 h after burying them at a 10 cm depth in a landfill for 144 h. Therefore, the biopolymers produced showed good properties such as flexibility, heat resistance and biodegradation. They have high potential and can be an alternative material to develop into food and drink packaging that is safe for humans and is also an environmentally friendly product.

## Figures and Tables

**Table 1:** Water solubility of bioplastic derived from water hyacinths, sugarcane bagasse and rice straw.

Type of Bioplastic	Rate of Water Solubility (%)		
	Glycerol Concentration (%*v*/*v*)		
	2	4	6
Water hyacinth	17.26	31.31	40.37
Bagasse	29.99	37.91	47.94
Rice straw	26.37	32.84	41.76

## Data Availability

The data that support the findings of this study are available from the corresponding author upon reasonable request.
